# Supplementary material for: Five Rice Seed-Specific NF-YC Genes Redundantly Regulate Grain Quality and Seed Germination via Interfering Gibberellin Pathway
Source: Int J Mol Sci. 2022 Jul 29;23(15):8382. doi: 10.3390/ijms23158382 (PMC9368926; doi:10.3390/ijms23158382)
Supplement: Supplementary file 1 [file ijms-23-08382-s001.zip › ijms-1765517-supplementary Figures S1-S5, Tables S1 and S2.pdf]

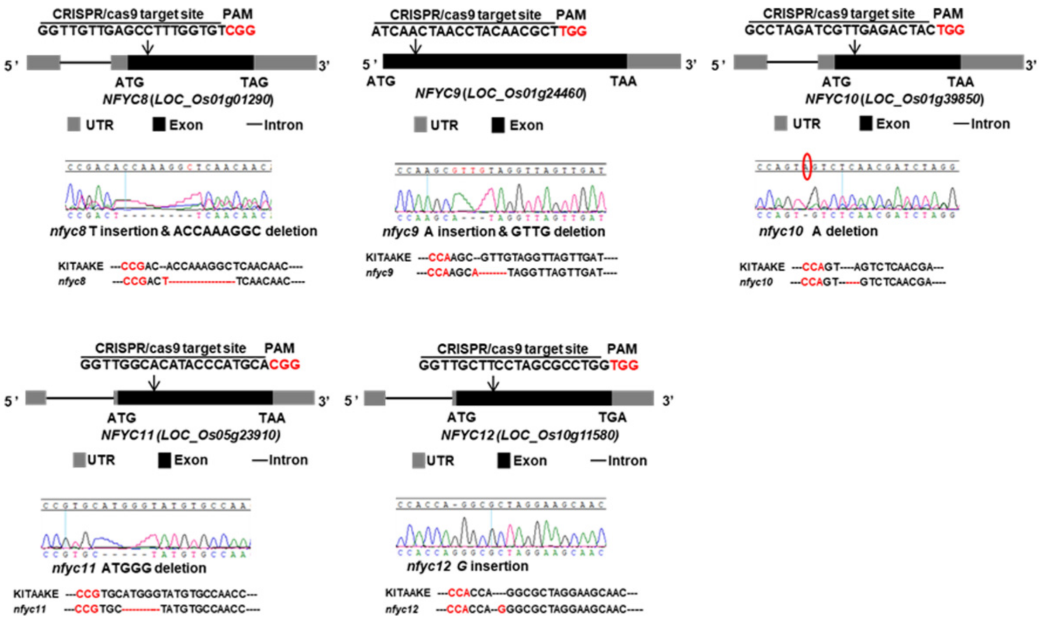

Figure S1 Genotyping of Kitaake and five *single-nfyc* lines. Sanger sequencing of the target sites in five single gene mutants of *nfyc8* to *nfyc12*. Red bases represent PAM, and red circles indicate insert or delete bases.

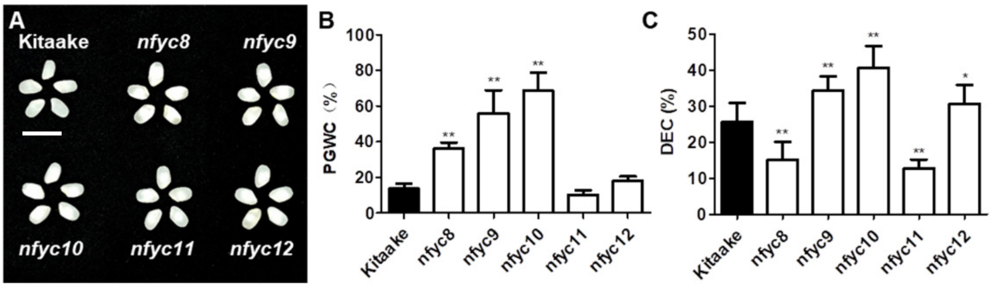

Figure S2 **A** Grain chalkiness phenotypical characterization of Kitaake and five single mutants, bar=1cm. **B.** Statistics of chalkiness grain rates of the five single mutants and Kitaake. **C.** Statistics of chalkiness degrees of the five single mutants and Kitaake. Bar=1cm.

Data are shown as means ± SD of at least three biological replicates. (*P < 0.05, **P < 0.01 by two-tailed Student's t-test).

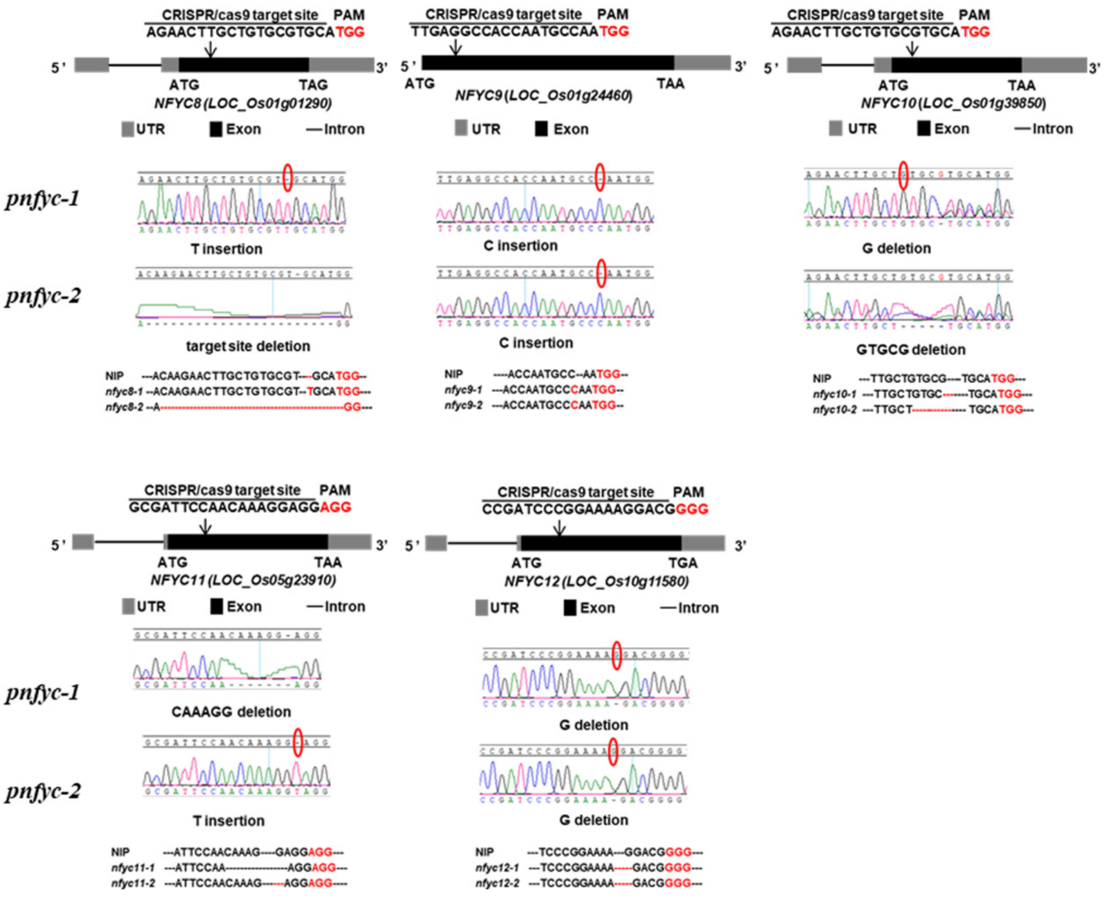

Figure S3 Genotyping of NIP and *pnfyc* lines. Sanger sequencing of the five target sites in *pnfyc (cr-nfyc8/yc9/yc10/yc11/yc12)* mutants showed two mutation types. Red bases represent PAM, and red circles indicate insert or delete bases.

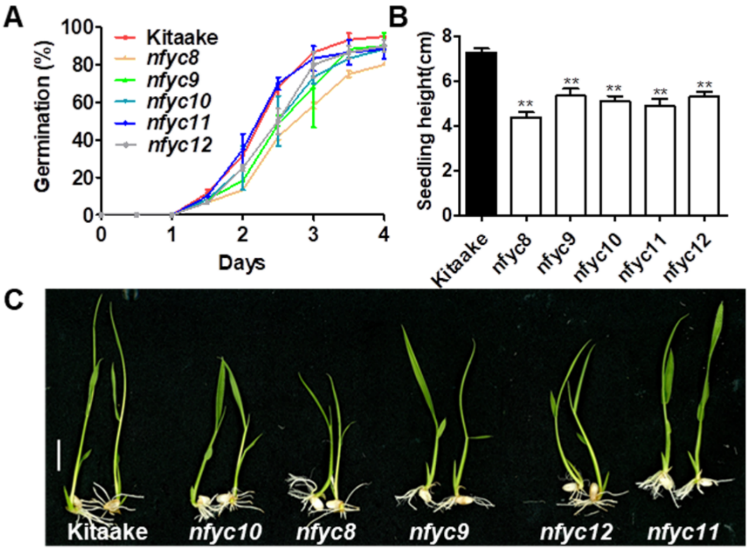

Figure S4 A The germination rate of Kitaake and five single mutants in 4 days. **B.** Statistics of the seedling height of Kitaake and five single mutants at 7 days after germination. **C.** The plant morphology of Kitaake and five single mutants at 7 days after germination. Bar=1cm.

Data are shown as means ± SD of at least three biological replicates. (*P < 0.05, **P < 0.01 by two-tailed Student's t-test).

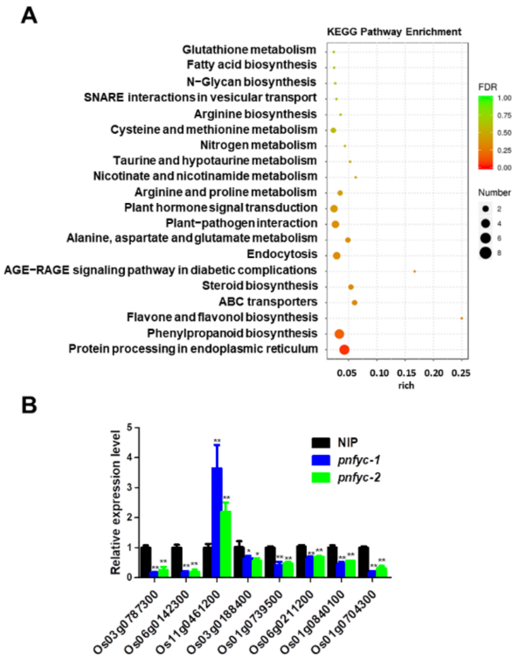

Figure S5 A One selection of KEGG pathway enrichment assay of the differentially expressed genes (DEGs) as determined by RNA-seq using embryos at 6 hours after germination. **B** qRT-PCR validation of the transcript levels of DEGs obtained from the RNA-seq results.

	Plant height (cm)	Panicle length (cm)	No.primary branch panicle	No.secondary branch panicle	No.effective panicles per plant	Seed setting rate(%)
NIP	99.07±4.31	21.44±1.34	11.56±1.78	16.55±6.78	8.40±2.48	86.41± 8.19
pnfyc-1	99.40±4.09	21.27±1.30	11.11±1.10	15.22±4.94	7.90±1.88	84.93±7.20
Pnfyc-2	97.20±2.66	21.38±1.80	11.21±1.20	15.11±5.11	7.50±1.61	84.05±7.75

Table S1 Quantification data of the panicle traits and plant height among NIP and *pnfyc* lines. Data are shown as means ± SD of at least three biological replicates. (*P < 0.05, **P < 0.01 by two-tailed Student's t-test).

	Onset temperature（℃）	Peak temperature（℃）	End set temperature（℃）
NIP	67.84±0.04	72.54±0.08	78.46±0.03
pnfyc-1	65.86±0.33 **	70.57±0.62*	76.61±0.14**
Pnfyc-2	65.77±0.22**	70.48±0.53*	76.12±0.21**

Table S2 Differential scanning calorimetry assay. Quantification data of gelatinization temperature among NIP and *pnfyc* lines. Data are shown as means ± SD of at least three biological replicates. (*P < 0.05, **P < 0.01 by two-tailed Student's t-test).
